# TSG-6 secreted by human adipose tissue-derived mesenchymal stem cells ameliorates severe acute pancreatitis via ER stress downregulation in mice

**DOI:** 10.1186/s13287-018-1009-8

**Published:** 2018-09-26

**Authors:** Qiang Li, Woo-Jin Song, Min-Ok Ryu, Aryung Nam, Ju-Hyun An, Jin-Ok Ahn, Dong Ha Bhang, Yun Chan Jung, Hwa-Young Youn

**Affiliations:** 1 Laboratory of Veterinary Internal Medicine, College of Veterinary Medicine, Seoul National University, 1 Gwanak-ro, Gwanak-gu, Seoul, 08826 Republic of Korea; 2 Department of Molecular and Cellular Biology, Samsung Biomedical Research Institute, Sungkyunkwan University School of Medicine, Suwon, Gyeonggi-do 16419 Republic of Korea; 3 BK21Plus program for 21st Century Biomedical Science Leader Development, Sungkyunkwan University School of Medicine, Suwon, Gyeonggi-do 16419 Republic of Korea; 4 Chaon, A-301-3, 240, Pangyoyeok-ro, Bundang-gu, Seongnam-si, Gyeonggi-do 13488 Republic of Korea

**Keywords:** Mesenchymal stem cells, Endoplasmic reticulum stress, NF-κB, TSG-6, Severe acute pancreatitis

## Abstract

**Background:**

Through recent studies, the onset of acute pancreatitis in pancreatic acinar cells (PACs) and the regulatory role of PACs in severe acute pancreatitis (SAP) have been revealed. During the early stages of pancreatitis, the endoplasmic reticulum (ER) in PACs undergoes significant changes, including swelling and vacuolization. In response to an increase in the extracellular stress in ER, PACs lose their functions, leading to cell apoptosis and inflammation response. The beneficial effects of human adipose tissue-derived mesenchymal stem cells (hAT-MSCs) on SAP have been well documented in previous studies. However, the underlying mechanism of their action remains controversial.

**Methods:**

In this study, the therapeutic effects of intraperitoneally administered hAT-MSCs in a caerulein (50 μg/kg)- and lipopolysaccharide (LPS) (10 mg/kg)-co-induced SAP mouse model were evaluated. Inflammatory response and ER stress were measured in pancreatic tissue samples, and the beneficial effects were evaluated through quantitative reverse transcription polymerase chain reaction (qRT-PCR), western blot, and immunofluorescence analysis.

**Results:**

Inflammatory response and ER stress were ameliorated following hAT-MSC injection, and the beneficial effects were observed in the absence of significant engraftment of hAT-MSCs. hAT-MSCs transfected with siRNA-targeting tumour necrosis factor-α-induced gene/protein 6 (TSG-6) were unable to inhibit ER stress and inflammation. In addition, TSG-6 from hAT-MSCs significantly suppressed ER stress-induced apoptosis and nuclear factor kappa B (NF-κB) activity in SAP model mice.

**Conclusions:**

TSG-6 secreted by hAT-MSCs protects PACs in SAP model mice via the inhibition of ER stress, as well as inflammatory responses. This study has revealed a new area for ER stress-targeted therapy in SAP patients.

**Graphical abstract:**

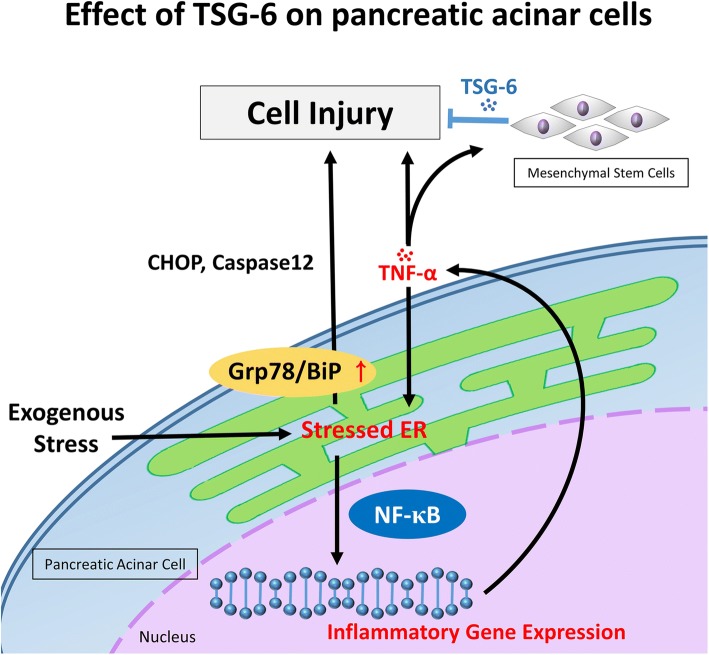

**Electronic supplementary material:**

The online version of this article (10.1186/s13287-018-1009-8) contains supplementary material, which is available to authorized users.

## Background

An increasing trend in the incidence of severe acute pancreatitis (SAP), an inflammatory disease known to cause a mortality rate as high as 43%, has been observed within the past decade [1]. The histopathological mechanism underlying SAP is not yet clear, and there is no specific or effective treatment for the disease [2]. Recent studies have revealed that the initiating events of SAP, including zymogen activation and cell death, occur in pancreatic acinar cells (PACs) [3]. Furthermore, the earliest immune response in acute pancreatitis was shown to originate within the PACs that activate circulating immune cells to induce a systemic inflammatory response [4].

The endoplasmic reticulum (ER) is the most important organelle in PACs; it is involved in the synthesis, folding, and maturation of secreted and transmembrane proteins [5]. ER stress response is a common cellular process; however, overactivation of ER stress may damage the cells through ER stress-induced apoptosis [6]. ER stress is caused by a variety of conditions, including calcium ion imbalance, oxidative stress, and inflammatory responses. Pro-inflammatory cytokine-induced ER stress may act as a critical factor during the early stages of SAP, and tumour necrosis factor alpha (TNF-α) is the most important pro-inflammatory cytokine that plays a central role in ER stress [7–9]. A high level of ER stress may result in the exacerbation of the inflammatory response in acute pancreatitis [10, 11].

Recent studies have demonstrated that mesenchymal stem cells (MSCs) ameliorate inflammatory diseases such as peritonitis, inflammatory bowel disease (IBD), diabetes, and pancreatitis [12–15]. The beneficial action of MSCs is associated with their homing effects and ability to repair tissues. Moreover, several studies have shown that MSCs may exert their effects without accumulating at the site of inflammation or tissue injury, suggestive of their actions through paracrine secretions. Furthermore, TNF-α-induced gene/protein 6 (TSG-6) has been shown to be the key secreted mediator of the anti-inflammatory response of MSCs in peritonitis, myocardial infarction, IBD, skin wound healing, and lung injury [16–20].

In this study, we investigated the effect of human adipose tissue-derived mesenchymal stem cells (hAT-MSCs) on caerulein- and lipopolysaccharide (LPS)-co-induced SAP mouse model. We specifically focused on ER stress-induced PACs after the administration of hAT-MSCs. As a result, we identified a systemic secretion of TSG-6 by hAT-MSCs to regulate ER stress and nuclear factor kappa B (NF-κB) signalling pathways.

## Methods

### Cell preparation

We obtained hAT-MSCs by the liposuction of the abdominal subcutaneous fat from a healthy donor after receiving written informed consent and according to the guidelines of the Institutional Review Board (IRB) of the R bio (IRB No. RBIO-2016-04-001). The cells were isolated and characterised as described in the Additional file 1: Methods, and the expression of stem cell markers was determined by flow cytometry (Additional file 2: Figure S1A). In addition, their differentiation abilities were confirmed through three-lineage stem cell differentiation kits (Invitrogen, Carlsbad, CA, USA), according to the manufacturer’s instructions (Additional file 2: Figure S1B). hAT-MSCs at passages 3–5 were used in the following experiments.

We separated PACs from C57BL/6 mice using collagenase digestion, as previously described [21, 22]. Upon the complete dissociation of the pancreatic tissue, the enzymatic reaction was stopped with the addition of cold HBSS (Invitrogen) supplemented with 5% foetal bovine serum (FBS; PAN Biotech, Aidenbach, Germany). The cell pellet was resuspended in modified PAC maintenance medium containing Dulbecco’s modified Eagle’s medium (DMEM)/F-12 (Invitrogen), 10% FBS (PAN Biotech), 1% penicillin-streptomycin (PAN Biotech), 0.25 mg/mL of soybean trypsin inhibitor (Sigma-Aldrich, St. Louis, MO, USA), and 10 ng/mL of recombinant human epidermal growth factor (Invitrogen), and incubated at 37 °C in a humidified atmosphere with 5% CO_2_ [23]. The isolated PACs were cultured for 24 h and characterised (Additional file 3: Figure S2A-C). Additionally, the viability of LPS- and caerulein-co-induced primary PACs were assessed using the D-plus^tm^ CCK kit (Dongyinbio, Seoul, Korea) at different time points, according to the manufacturer’s instructions (Additional file 3: Figure S2D).

### Animals

Male C57BL/6 mice weighing approximately 20–25 g were purchased from the Central Lab Animal Inc. (Seoul, Korea). All mice were housed in a specific pathogen-free standard room under controlled conditions of temperature (20–22 °C), humidity (50% ± 5%), and light cycle (12:12 h light-dark). The study and all experimental procedures involving animals were approved by the institutional Animal Care and Use Committee of Seoul National University (SNU-180410-4), and the experiments were performed as per the Guidelines for animal experiments. Experimental mice were fasted for 12 h before treatment; they had free access to water.

### Transfection of hAT-MSCs with TSG-6 siRNA

hAT-MSCs were plated in six-well plates and 40–50% confluent cells were transfected with TSG-6 siRNA (sc-3981; Santa Cruz Biotechnology, Dallas, TX, USA) or scrambled (scr) siRNA (sc-37007; Santa Cruz Biotechnology) for 48 h using Lipofectamine RNAiMAX (Invitrogen), according to the manufacturer’s instructions. TSG-6 knockdown in these cells was confirmed using quantitative reverse transcription polymerase chain reaction (qRT-PCR) before their application for further studies.

### Induction and treatment of SAP

For the induction of the SAP model, mice were administered with an hourly intraperitoneal injection of caerulein (50 μg/kg, six times; Sigma-Aldrich) and LPS (10 mg/kg; Sigma-Aldrich), as previously described [24]. To evaluate the therapeutic efficacy, mice treated once with or without hAT-MSCs (1 × 10^6^ cells/per mouse) were sacrificed 12, 24, or 48 h after hAT-MSC infusion. Additionally, SAP mice were randomised into the phosphate-buffered saline (PBS)-treated, hAT-MSC-treated, scr siRNA-transfected hAT-MSC-treated, or TSG-6 siRNA-transfected hAT-MSC-treated groups (*n* = 8, in each group). The mice were administered with 200 μL of PBS after LPS infusion. In each experiment, naive mice were treated with PBS alone or PBS in combination with hAT-MSCs as the sham and MSC sham groups, respectively (*n* = 5, in each group). The mice were sacrificed, and the serum and pancreatic tissues were collected for further analysis.

### Assessment of pancreatitis severity

To assess the severity of pancreatitis and choose an appropriate time point for further evaluation, parameters for acute pancreatitis, including pancreatic oedema (pancreas/body weight ratio), and serum amylase and lipase activities were measured 12, 24, and 48 h after hAT-MSC administration. Amylase and lipase activities were evaluated using the mouse pancreatic amylase enzyme-linked immunosorbent assay (ELISA) kit (Cusabio Biotech Co, Ltd., Wuhan, China) and pancreatic lipase ELISA kit (Mybiosource, San Diego, CA, USA), respectively.

### Histological examination

Pancreas and lung tissues were rinsed and fixed in 4% paraformaldehyde, embedded in paraffin, and cut into 4-μm sections that were stained with haematoxylin and eosin (H&E). To quantify pancreatic tissue injury, 10 random fields per group were evaluated by two investigators in a blinded manner under a light microscope (magnification, ×200 or ×400) using a modified evaluation system, as described in Additional file 4: Table S1 [25]. In addition, pancreatitis-associated lung injury was evaluated as per the previous report (Additional file 5: Figure S3) [26].

### Co-culture of isolated PACs and hAT-MSCs

All co-culture experiments were conducted in six-well cell culture plates containing 0.4-μm pore-sized transwell inserts (SPL Life Science, Pocheon, Korea). A total of 3 × 10^5^ hAT-MSCs, scr siRNA-transfected hAT-MSCs, or TSG-6 siRNA-transfected hAT-MSCs were plated at the bottom of the six-well plates (SPL Life Science). After the attachment of cells to the plates, isolated PACs were seeded onto the transwell inserts and stimulated with 100 nM caerulein (Sigma-Aldrich) and 10 mg/mL LPS (Sigma-Aldrich) for 12 h. The co-culture system was maintained at 37 °C in a humidified atmosphere with 5% CO_2_. Naive PACs and non-hAT-MSCs treated with caerulein and LPS served as the naive and positive control groups, respectively.

### Quantitative reverse transcription polymerase chain reaction (qRT-PCR) analysis

Total RNA was extracted from the homogenised pancreatic tissue, isolated PACs, and hAT-MSCs using the Easy-BLUE Total RNA Extraction kit (Intron Biotechnology, Seongnam, Korea), according to the manufacturer’s instructions. cDNA was synthesised from 1 μg of total RNA with CellScript All-in-One cDNA Master Mix (CellSafe, Yongin, Korea), and the samples were analysed in triplicate using AMPIGENE qPCR Green Mix Hi-ROX with the SYBR Green dye (Enzo Life Sciences, Farmingdale, NY, USA). The expressions of target genes were analysed according to the 2^−ΔΔ/Cts^ method and normalised to the mRNA levels of glyceraldehyde-3-phosphate dehydrogenase (GAPDH). The primer sequences used in this study are listed in Additional file 4: Table S2.

### Western blot analysis

The total protein from pancreatic tissue and isolated PACs was extracted in PRO-PREP Protein Extraction Solution (Intron Biotechnology) on ice according to the manufacturer’s instructions, and the protein concentrations were measured using the Bio-Rad DC Protein Assay Kit (Bio-Rad, Hercules, CA, USA). A total of 20 μg of each of the proteins was subjected to electrophoresis on 10% sodium dodecyl sulphate-polyacrylamide gels, and the protein bands were transferred onto polyvinylidene difluoride membranes (EMD Millipore, Billerica, MA, USA). The membranes were incubated in 5% non-fat dry milk in Tris-buffered saline containing 0.1% Tween-20 for 1 h and treated with antibodies against anti-caspase-12 (1:500), anti-CCAAT-enhancer-binding protein homologous protein (CHOP) (1:500), anti-78-kDa glucose-regulated protein (Grp78) (1:1000) (all from Cusabio Biotech Co), anti-TNF-α (1:1000), anti-NF-κB-p65 (1:1000), and anti-NF-κB-p-p65 (1:1000) (Cell Signaling Technology, Beverly, Massachusetts, USA) at 4 °C overnight. The membranes were incubated with anti-rabbit or anti-mouse IgG (Santa Cruz Biotechnology) as the secondary antibodies (1:2000) for 1 h. The protein bands were visualised using enhanced chemiluminescence (Advansta, Menlo Park, CA, USA) and normalised to the levels of β-actin (Santa Cruz Biotechnology).

### Quantification of cell apoptosis

Cell death was analysed for PACs treated with LPS plus caerulein. Paraffin-embedded pancreatic tissue sections were deparaffinised in xylene and rehydrated with ethanol solution. Apoptosis rates were determined by TUNEL staining (Apo-BrdU DNA Fragmentation Assay Kit; BioVision, San Francisco, USA), following the manufacturer’s instructions. Immunoreactive cells were counted in 10 random fields per group using an EVOS FL microscope (Life Technologies, Darmstadt, Germany).

### Standard curve of human GAPDH

Standard curves were generated by adding serial dilutions of hAT-MSCs to mouse tissues, including those of the heart, liver, lung, kidney, spleen, brain, and pancreas. 100–100,000 stem cells were added to the whole mouse organs prior to homogenisation and total RNA was extracted using the Easy-BLUE Total RNA Extraction kit (Intron Biotechnology). cDNA was generated (CellScript All-in-One cDNA Master Mix, CellSafe) using 1 μg of total RNA. The synthesized cDNA was analysed by qRT-PCR with human-specific GAPDH, and the final value for total cDNA in the sample was corrected by parallel qRT-PCR assays with primers that amplified both the human/mouse genes for GAPDH, as previously described [17, 27].

### Statistical analysis

Data are shown as mean ± standard deviation. Differences between groups were compared by one-way analysis of variance or Student’s *t* test with GraphPad Prism v.6.01 software (GraphPad Inc., La Jolla, CA, USA). *P* < 0.05 was considered as statistically significant.

## Results

### Intraperitoneally administered hAT-MSCs ameliorated SAP

**Fig. 1:**
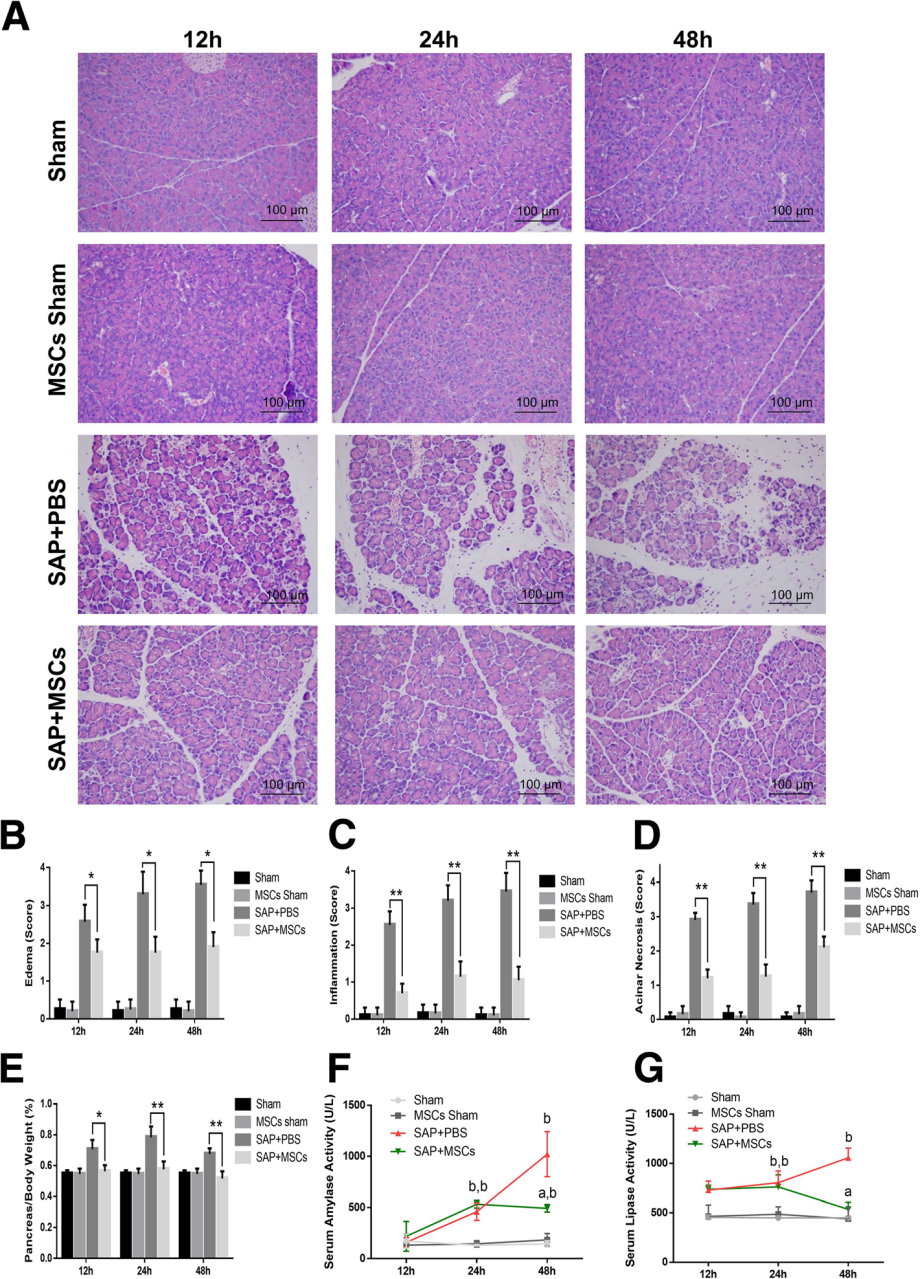
Effects of intraperitoneally injected hAT-MSCs on caerulein- and LPS-co-induced severe acute pancreatitis (SAP). Mice were treated with caerulein (50 μg/kg, six times) and LPS (10 mg/kg) to induce SAP. Mice were sacrificed 12, 24, and 48 h after hAT-MSCs infusion. **a** Representative pathological changes in H&E-stained pancreatic tissue sections at ×200 magnification. **b**, **c**, **d** Histological analysis of the severity of acute pancreatitis. **e** The ratio of pancreas weight to body weight was measured. **f**, **g** The activities of digestive enzymes such as serum amylase and lipase were measured. Each value represents the mean ± SD. ^*^P < 0.05, ^**^P < 0.01, ^a^*P* < 0.01 versus SAP + PBS group at the same time point; ^b^*P* < 0.01 versus MSC sham group at the same time point (*n* = 5–8 mice per group), *MSC* mesenchymal stem cells, *PBS* phosphate-buffered saline, *SAP* severe acute pancreatitis

To determine the effect of hAT-MSCs on SAP, we treated mice with hAT-MSCs and the treatment effects were evaluated 12, 24, and 48 h after hAT-MSC infusion. Histological examination of the pancreatic tissues after the induction of SAP revealed tissue injury characterised by tissue oedema, acinar cell necrosis, and inflammatory cell infiltration (Fig. 1a). In comparison with the SAP + PBS group, the SAP + MSC group showed a significant reduction in the pancreatic tissue injury at the 12, 24, and 48 h time points (Fig. 1b, c, d). Pancreatic oedema was evaluated based on the pancreas to body weight ratio. SAP models showed a significant increase in the pancreas to body weight ratio, while hAT-MSC treatment attenuated these effects at the 12, 24, and 48 h time points (Fig. 1e). The analysis of the serum pancreatic enzyme activity showed that hAT-MSC administration markedly decreased the activities of serum amylase and lipase at the 48 h time point (Fig. 1f, g).

### hAT-MSCs reduced the inflammatory response and ER stress in pancreatic tissues

**Fig. 2:**
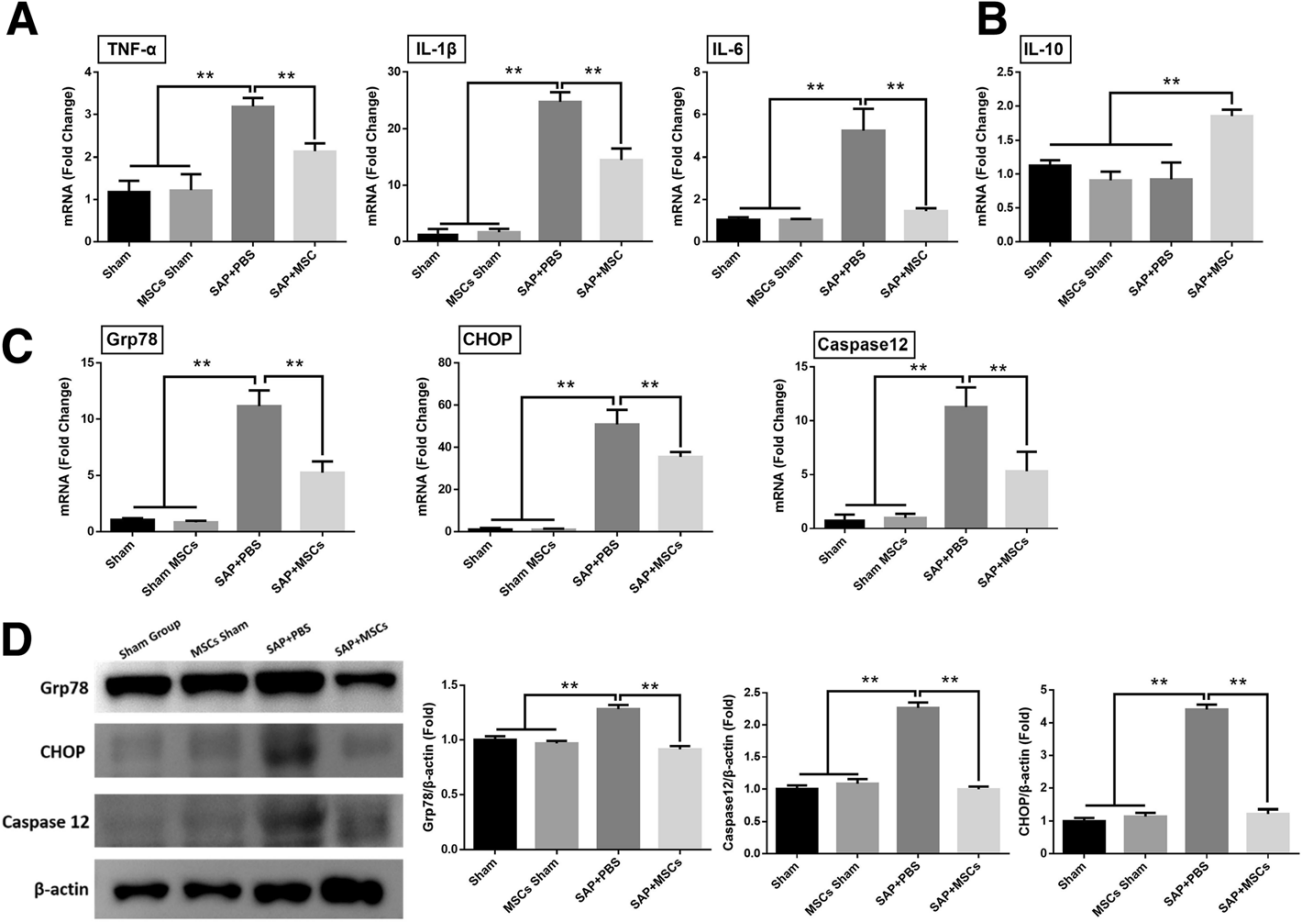
Effects of hAT-MSCs on inflammatory cytokine and ER stress levels. **a**, **b** The mRNA expression levels of inflammatory cytokines in the pancreatic tissue at the 48 h time point. **c** mRNA and (**d**) protein expression levels of Grp78, CHOP, and caspase-12 in the pancreatic tissue 48 h after the administration of hAT-MSCs. Results are presented as the mean ± SD obtained from three independent experiments. ^*^P < 0.05, ^**^P < 0.01. *CHOP* CCAAT-enhancer-binding protein homologous protein, *Grp7* 78-kDa glucose-regulated protein, *IL* interleukin, *MSC* mesenchymal stem cells, *PBS* phosphate-buffered saline, *SAP* severe acute pancreatitis, *TNF-α* tumour necrosis factor alpha

To determine whether hAT-MSCs regulate inflammatory response under conditions of inflammation, the mRNA levels of inflammatory cytokines in the pancreatic tissue were measured by qRT-PCR analysis. The expression levels of pro-inflammatory cytokines (TNF-α, interleukin [IL]-1β, and IL-6) were markedly increased in SAP + PBS group but significant decreased in the group treated with hAT-MSCs (Fig. 2a). While the mRNA level of the anti-inflammatory cytokine IL-10 failed to increase in the SAP + PBS group, it was significantly elevated in the SAP + MSC group (Fig. 2b). In addition, we evaluated the expression levels of the relative markers of ER stress (Grp78, CHOP, and caspase-12) by qRT-PCR and western blot analyses, and found that the hAT-MSC-treated group showed a marked decrease in the mRNA levels of Grp78, CHOP, and caspase-12, compared to the case in the SAP + PBS group. Similar results were observed for protein analysis, wherein a significant decrease in ER stress-related marker levels was observed in the SAP + MSC group, compared to the case in the SAP + PBS group (Fig. 2c, d).

### Intraperitoneally injected hAT-MSCs did not accumulate in the pancreatic tissues

**Fig. 3:**
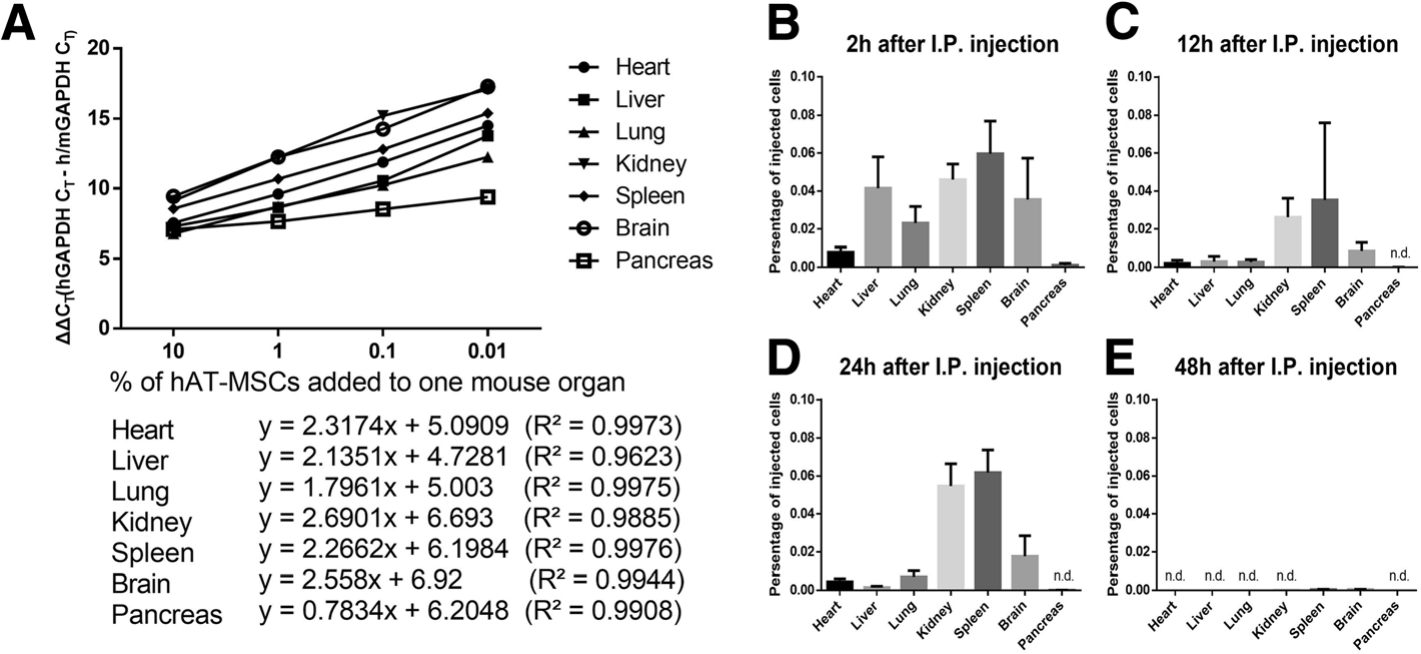
Assay for evaluating the fate of intraperitoneally injected hAT-MSCs. A serial dilution of 1 × 10^6^ hAT-MSCs was injected in mice to investigate the expression level of human/mouse GAPDH and human-specific GAPDH. **a** Standard curves for quantitative reverse transcription polymerase chain reaction assay of human-specific mRNA levels of GAPDH. **b**, **c**, **d**, **e** Distribution of hAT-MSCs in seven organs 2, 12, 24, and 48 h after their intraperitoneal infusion. The results are representative of three independent experiments. *GAPDH* glyceraldehyde-3-phosphate dehydrogenase, *hAT-MSCs* human adipose tissue-derived mesenchymal stem cells, *I.P.* intraperitoneal

To detect the fate of hAT-MSCs infused intraperitoneally into SAP mice, we quantified the injected hAT-MSCs (1 × 10^6^ cells) by constructing standard curves using qRT-PCR (Fig. 3a). After 2 h of administration, the percentages of hAT-MSCs detected in the heart, liver, lung, kidney, spleen, brain, and pancreas of SAP mice were 0.079%, 0.415%, 0.023%, 0.046%, 0.059%, 0.035%, and 0.001%, respectively (Fig. 3b). After 12 to 24 h, no hAT-MSCs were detected in the pancreatic tissue (Fig. 3c, d). At the 48 h time point, a few hAT-MSCs were observed in the spleen and brain tissues, but not in other tissues (Fig. 3e).

### TSG-6 secreted by hAT-MSCs alleviated the ER stress levels in PACs in vitro

**Fig. 4:**
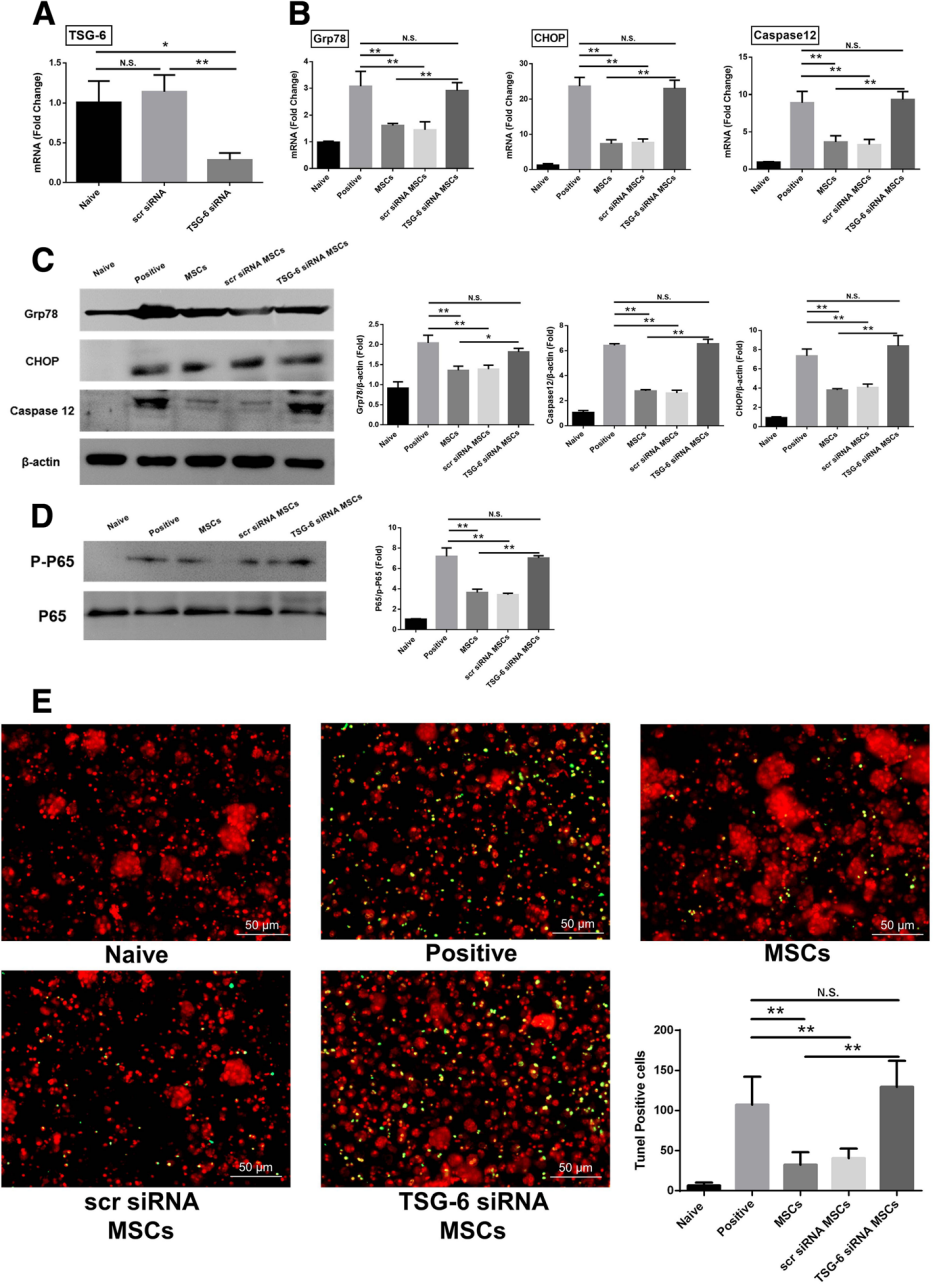
In vitro effect of TSG-6 on caerulein- and LPS-stimulated primary pancreatic acinar cells (PACs). **a** TSG-6 mRNA expression levels in hAT-MSCs transfected with TSG-6 siRNA (TSG-6 siRNA MSCs), hAT-MSCs transfected with control siRNA (scr siRNA MSCs), and naive hAT-MSCs (MSCs). **b**, **c** Caerulein- and LPS-stimulated primary PACs were indirectly co-cultured with each of the three hAT-MSC types for 12 h. **b** Grp78, CHOP, and caspase-12 mRNA expression levels in primary PACs. **c** Grp78, CHOP, and caspase-12 protein expression levels. **d** The expression levels of NF-κB p65 activity were analysed by western blotting. **e** TUNEL staining in PACs in each group observed at ×200 magnification. TUNEL staining revealed the number of cells undergoing apoptosis or pyroptosis. Results are presented as the mean ± SD obtained from three independent experiments ^*^P < 0.05, ^**^P < 0.01. *CHOP* CCAAT-enhancer-binding protein homologous protein, *Grp7* 78-kDa glucose-regulated protein, *MSC* mesenchymal stem cells, *PBS* phosphate-buffered saline, *SAP* severe acute pancreatitis, *scr* scrambled, *TSG-6* tumour necrosis factor-α-induced gene/protein 6

To evaluate the effects of TSG-6 secreted by hAT-MSCs on ER stress, hAT-MSCs were transduced with TSG-6 siRNA or scr siRNA and the gene expression of TSG-6 was measured by qRT-PCR analysis. hAT-MSCs transfected with TSG-6 siRNA showed a significant decrease in the expression of the TSG-6 mRNA level, whereas no significant change in the TSG-6 mRNA level was observed in hAT-MSCs transfected with control siRNA (scr siRNA), compared to the case for naive hAT-MSCs (Fig. 4a).

hAT-MSCs transfected with TSG-6 siRNA showed no change in the differentiation potential in vitro (Additional file 1: Figure S1B). We co-cultured caerulein- and LPS-stimulated PACs with naive or siRNA-transfected hAT-MSCs in a transwell system for 12 h and found that the expression levels of Grp78, CHOP, and caspase-12 were significantly decreased in the hAT-MSC- and scr siRNA-transfected hAT-MSC-treated groups, compared to the case for the positive control and TSG-6 siRNA-transfected hAT-MSC-treated groups (Fig. 4b). However, no significant change was observed in the expression levels of ER stress markers in the group treated with TSG-6 siRNA-transfected hAT-MSCs, compared to the positive control group (Fig. 4c). NF-κB is related with ER stress-induced apoptosis and inflammatory response as a multi-functional transcript factor. To determine the relationship between TSG-6 and NF-κB in PACs, we measured the NF-κB activity by evaluating the ratio of phosphorylated P56 to P56. NF-κB activity was significantly suppressed in the hAT-MSC- and scr siRNA-transfected hAT-MSC-treated groups, compared to the case in the positive control and TSG-6 siRNA-transfected hAT-MSC-treated groups (Fig. 4d).

To determine whether TSG-6 regulates PAC apoptosis, we quantified the PAC apoptotic ratio using the TUNEL assay. The results showed that treatment with naive hAT-MSCs or hAT-MSCs transduced with scr siRNA may significantly reduce the apoptotic ratio, compared to the case in the positive control group; however, we failed to observe similar effects with hAT-MSCs transduced with TSG-6 siRNA.

### Knockdown of TSG-6 in hAT-MSCs failed to ameliorate SAP

**Fig. 5:**
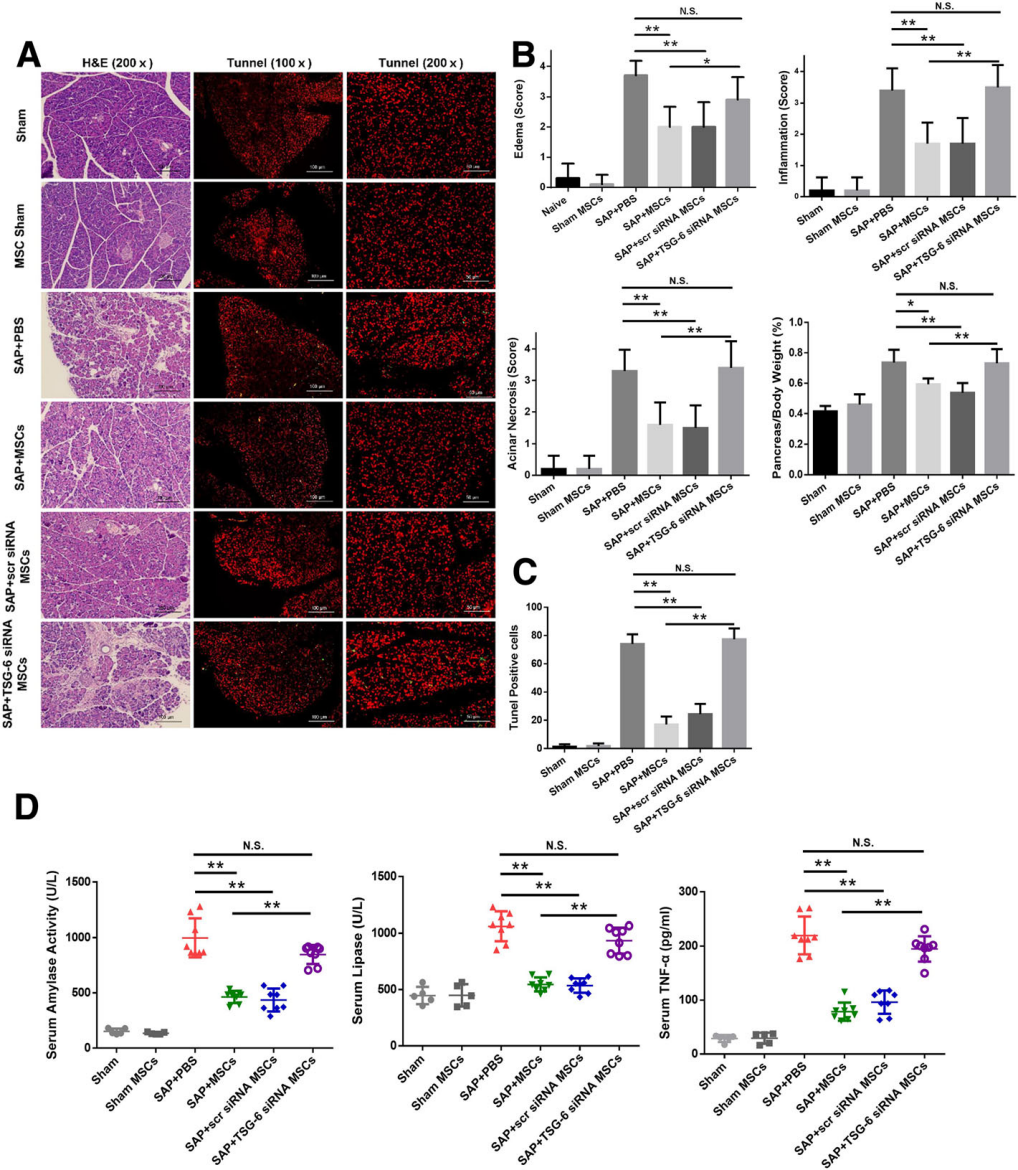
Effects of TSG-6 knockdown in hAT-MSCs on caerulein and LPS co-induced SAP. **a** Representative H&E and TUNEL staining images of the pancreatic tissue sections in each group at 200× magnification. TUNEL staining revealed the number of cells undergoing apoptosis or pyroptosis. **b** The histological scores of H&E and pancreas/body weight ratio were evaluated. **c** TUNEL-positive cells were counted for each group at 200× magnification. **d** Collected serum samples were analysed by ELISA for the evaluation of amylase activity, lipase activity, and TNF-α concentration. Results are presented as the mean ± SD. ^*^P < 0.05, ^**^P < 0.01 (n = 5–8 mice per group). *MSC* mesenchymal stem cells, *PBS* phosphate-buffered saline, *SAP* severe acute pancreatitis, *scr* scrambled, *TSG-6* tumour necrosis factor-α-induced gene/protein 6

We evaluated whether hAT-MSCs transduced with TSG-6 siRNA ameliorate SAP. In comparison with the PBS-treated group, the groups treated with hAT-MSCs and scr siRNA-transfected hAT-MSCs showed a significant reduction in the degree of pancreatic tissue oedema, acinar cell necrosis, and inflammatory cell infiltration. We failed to observe this effect in the group treated with TSG-6 siRNA-transfected hAT-MSCs (Fig. 5a, b). TUNEL staining was performed on the pancreatic tissue sections, and the result showed that the number of TUNEL-positive cells was significantly reduced after treatment with hAT-MSCs or scr siRNA-transfected hAT-MSCs; no significant improvement in apoptosis was observed for the group treated with TSG-6 siRNA-transfected hAT-MSCs and PBS (Fig. 5c). In comparison with the PBS-treated group, the groups treated with hAT-MSCs and scr siRNA-transfected hAT-MSCs showed a significant reduction in the levels of serum amylase and lipase activities, and TNF-α concentration (Fig. 5d). On the contrary, no effect on SAP was seen in the group treated with TSG-6 siRNA-transfected hAT-MSCs.

**Fig. 6:**
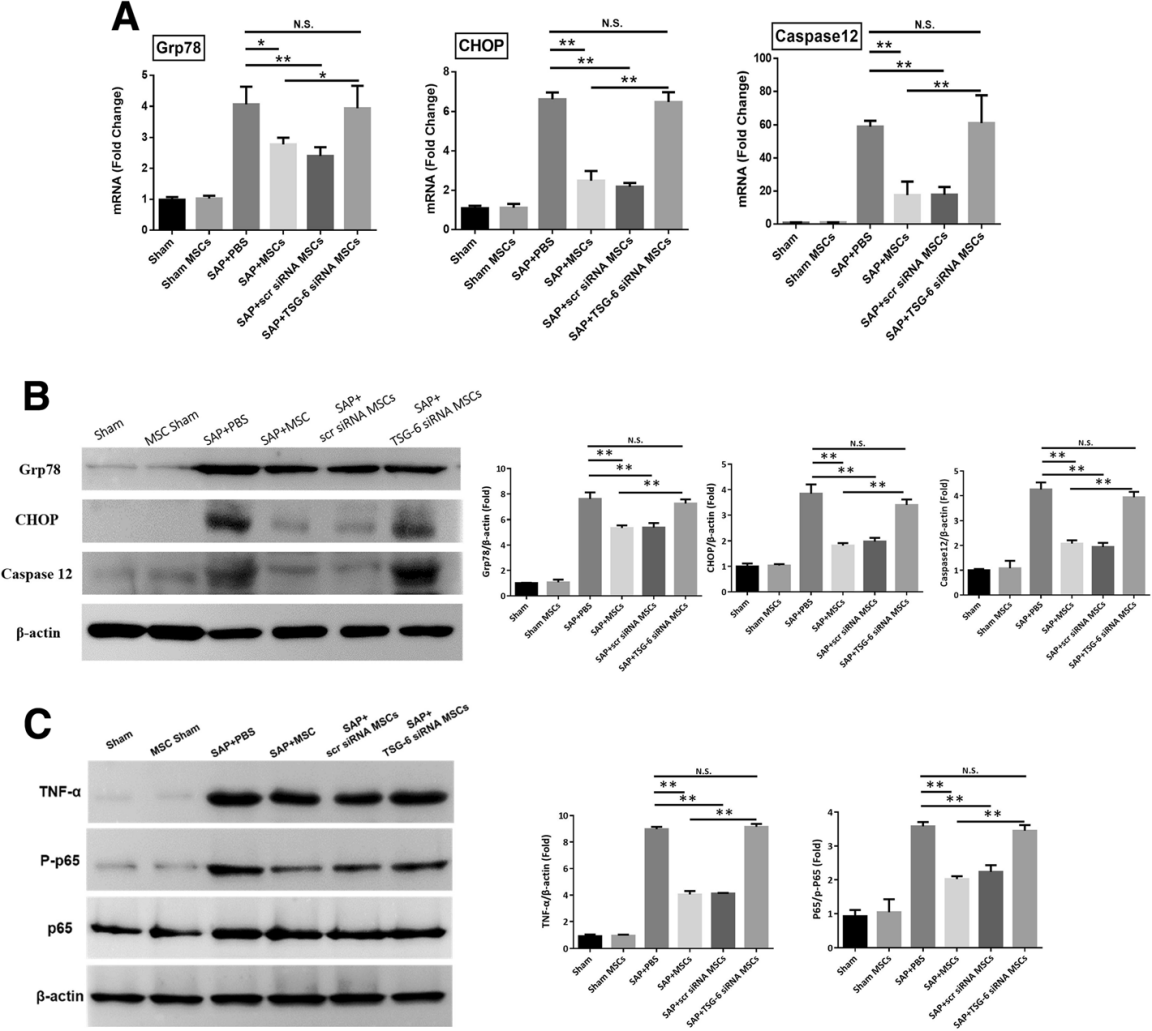
Beneficial effects of hAT-MSCs depend on TSG-6 secretion due to ER stress and NF-κB activity. **a**, **b** The mRNA and protein expression levels of Grp78, CHOP, and caspase-12 were analysed by qRT-PCR and western blotting, respectively. The band intensity on films is presented as the relative ratio of Grp78, CHOP, and caspase-12 to the band intensity of β-actin. **c** The expression levels of TNF-α and activity of p65 were analysed by western blotting. The band intensity on films is presented as the relative ratio of the level of TNF-α to that of β-actin; for NF-κB signalling, p65 activity was presented in the form of the ratio of phosphorylated p65 to p65. Results are presented as the mean ± SD obtained from three independent experiments ^*^P < 0.05, ^**^P < 0.01. *CHOP* CCAAT-enhancer-binding protein homologous protein, *Grp7* 78-kDa glucose-regulated protein, *MSC* mesenchymal stem cells, *PBS* phosphate-buffered saline, *SAP* severe acute pancreatitis, *scr* scrambled, *TSG-6* tumour necrosis factor-α-induced gene/protein 6

We assessed the expression level of ER stress-related markers in the pancreatic tissues of SAP mice. Intraperitoneal administration of hAT-MSCs transfected with TSG-6 siRNA failed to reduce the expression levels of ER stress-related markers at both the mRNA and protein levels (Fig. 6a, b). We investigated the protein levels of p65 and phosphorylated p65 in the pancreatic tissue by western blot analysis, to evaluate whether TSG-6 could modulate NF-κB signalling in vivo. In addition, we measured local TNF-α protein expression levels in the pancreatic tissues (Fig. 6c). As a result, we found that the groups treated with hAT-MSCs or hAT-MSCs transfected with scr siRNA showed a significant reduction in the expression levels of TNF-α and NF-κB activity in the pancreatic tissues, while this effect was absent in groups treated with TSG-6 siRNA-transfected hAT-MSCs and PBS.

## Discussion

Recent studies have shown the anti-inflammatory effects of hAT-MSCs on SAP; however, their mechanism of action is still unclear. In the present study, we found that the intraperitoneally administered hAT-MSCs ameliorated caerulein- and LPS-co-induced SAP in the absence of any significant engraftment of the hAT-MSCs. Our study also indicated that TSG-6, as the multifunctional protein with anti-inflammatory action, was secreted by hAT-MSCs in response to the inflammatory environment and ER stress. These results suggest that hAT-MSCs exert their effects on PACs via the downregulation of inflammatory response and ER stress during the early stages of SAP.

Several studies have demonstrated that SAP may be induced by various reagents. Mice treated with caerulein alone exhibited pancreatic inflammation, and hence, this model is suitable to study mild acute pancreatitis and is widely used for the analysis of intracellular changes during the early phase of acute pancreatitis [28]. The combination treatment with caerulein and LPS to induce SAP in mice results in easily to practice and the same pathological signs of human SAP, including pancreatic necrosis, oedema, and inflammation [24, 29]. To study the therapeutic effects of hAT-MSCs, caerulein- and LPS-induced SAP mice were evaluated 12, 24, and 48 h after hAT-MSC administration. The histopathological changes in pancreatitis scores significantly improved after one hAT-MSC treatment for 12, 24, and 48 h; however, serum amylase and lipase activities were primarily detected at the 48 h time point. We concluded that hAT-MSCs are potentially capable of restricting SAP-induced pancreatic injury through the inhibition of PAC injury during the early stages. In addition, hAT-MSCs administration leads to the timely reduction in pancreatic damage and pancreatitis-associated lung injury. Thun, hAT-MSCs offer protection against the development of pancreatitis and systemic inflammation, which may cause the perpetuation of a vicious cycle.

PACs are rich in ER, which performs the function of synthesising digestive enzymes. These cells are more prone to damage caused by external stimuli, thus leading to ER stress [30]. During the early stages of pancreatitis, the ER structure undergoes significant changes, including swelling and vacuolisation [31, 32]. Under normal conditions, ER stress sensors (inositol-requiring enzyme [IRE]-1α, protein kinase RNA [PKR]-like ER kinase [PERK], and activating transcription factor [ATF6]) are maintained in their inactive states through binding to the ER chaperone BiP (also known as Grp78). Both CHOP and caspase-12 are known as key factors that contribute to ER stress-induced apoptosis [11, 33, 34]. ER stress damages the acinar cell functions and induces apoptosis and inflammation response via ER stress and NF-κB signalling pathways [35–37]. In the present study, we evaluated whether the administered hAT-MSCs simultaneously regulate the inflammatory response and ER stress. As shown in Fig. 2, the levels of inflammatory cytokines (TNF-α, IL-1β, and IL-6) and ER stress-related markers (Grp78, CHOP, and caspase-12) were increased after caerulein and LPS stimulation in the pancreatic tissue at the 48 h time point, and hAT-MSC administration significantly reduced the levels of inflammatory cytokines and ER stress-related markers.

It is interesting to study the fate of MSCs after their intraperitoneal injection. It was demonstrated that intraperitoneally injected stem cells quickly form clusters with the resident innate immune cells in the peritoneal cavity and the resulting aggregation probably limits the access of stem cells into the systemic circulation [38]. We studied the distribution of intraperitoneally injected hAT-MSCs by qRT-PCR. After 2 h of the hAT-MSC administration, less than 1% of the total injected cells were detected in the heart, liver, lung, kidney, spleen, brain, and pancreas of SAP mice. Furthermore, we failed to detect hAT-MSCs in the pancreas after 12 h, while the number of hAT-MSCs in other tissues was negligible after 48 h.

Stem cells regulate the inflammatory response via the secretion of several molecules, including TGF-β, indoleamine-2,3-dioxygenase, and prostaglandin E2 (PGE2) [39, 40]. In addition, the therapeutic effects of MSCs were shown to be related to the expression level of TSG-6 [16, 20, 41–43]. Our recent study indicated that the upregulation of TSG-6 expression in TNF-α-pre-conditioned hAT-MSCs significantly improved the therapeutic effects in IBD models [44]. Based on these results and our previous study, it is tempting to speculate if TSG-6 plays a critical role in SAP.

In this study, we constructed hAT-MSCs negative for TSG-6 expression to evaluate the levels of ER stress markers in PACs. The indirect co-culture of caerulein- and LPS-stimulated primary PACs and naive or siRNA-transfected hAT-MSCs in a transwell system showed that the levels of ER stress-related markers significantly increased in the positive control group and the group treated with TSG-6 siRNA-transfected hAT-MSCs, compared to the case in the groups treated with naive hAT-MSCs and scr siRNA-transfected hAT-MSCs. We measured the ratio of ER stress-induced apoptotic cells in vitro and found that the ratio decreased after the treatment with naive hAT-MSCs or hAT-MSCs transduced with scr siRNA; we failed to observe similar effects after treatment with TSG-6 siRNA-transfected hAT-MSCs. Our data suggest that TSG-6 plays a critical role in the alteration of the expression of ER stress-related markers and apoptotic ratio in PACs via a distant, indirect action.

Based on the aforementioned results, we evaluated the effects of TSG-6 released from hAT-MSCs in vivo. In comparison with SAP mice treated with PBS, those treated with TSG-6 siRNA-transfected hAT-MSCs showed no significant improvement in the histopathological score, and no significant reduction in the apoptotic ratio was observed. Serum amylase and lipase activities serve as clinical markers of acute pancreatitis and reveal the absence of any injury [45]. No significant reduction in these enzyme activities was observed in mice treated with TSG-6 siRNA-transfected hAT-MSCs. TNF-α is the most elevated cytokine, which plays a central role in SAP and mediates the early phase of inflammation and ER stress-related molecular activation [46]. The expression levels of TNF-α in serum and pancreatic tissue were measured by ELISA and western blotting, respectively. The administration of hAT-MSCs significantly reduced the expression levels of TNF-α in the serum and pancreatic tissue, while no change in the expression levels was observed after treatment with TSG-6 siRNA-transfected hAT-MSCs. Collectively, these results indicate that TSG-6 from hAT-MSCs inhibits the systemic and local TNF-α expression.

The NF-κB signalling pathway in PACs plays a vital and initial role in the induction of inflammatory cytokine expression [47] and ER stress-induced apoptosis [48–50]. Furthermore, NF-κB acts as an important link between ER stress and inflammatory response [51, 52]. The previous study has been revealed that therapeutic effects of TSG-6 were depended on CD44 expression by PACs and significantly inhibition NF-κB activation [41]. In the present study, we analysed the ratio of phosphorylated p65 to p65, and found a significant reduction in this ratio in the groups treated with naive hAT-MSCs and scr siRNA-transfected hAT-MSCs. This effect was absent in both the pancreatic tissue and PACs in the groups treated with TSG-6 siRNA-transfected hAT-MSCs.

There are some limitations in this study. Recent studies have showed adult stem cells from different donors varied widely in their efficacy, and the ability maybe influenced by various factors, including age and physiological status [53, 54]. Therefore, further studies using hAT-MSCs from different donors might help predict efficacy of stem cell therapy in pancreatitis patients. Also, it is possible that one or more factors from hAT-MSCs may contribute to the suppression of ER stress-induced apoptosis. Hepatocyte growth factor secreted by bone marrow-derived mesenchymal stem cells was shown to reduce ER stress in alveolar epithelial cells [55]. Further studies are warranted to investigate if NF-κB inhibitors affect the ER stress pathway after hAT-MSC administration. Above all, TSG-6 plays an important role in ER stress-induced apoptosis and early phase of inflammatory response, probably by reducing the ER stress and inhibiting the NF-κB pathway in PACs.

## Conclusions

In summary, we showed that intraperitoneally administered hAT-MSCs significantly alleviated inflammation from pancreatic injury in an SAP mouse model. The beneficial effects of hAT-MSCs were demonstrated with the absence of any significant engraftment of hAT-MSCs in the injured pancreatic tissue. These effects were dependent on TSG-6, a critical anti-inflammatory cytokine secreted after TNF-α stimulation. Furthermore, TSG-6 secreted from hAT-MSCs significantly suppressed ER stress-induced apoptosis and NF-κB activity in PACs during the early stage of SAP. These findings may facilitate the development of MSC-based therapeutic strategies for the treatment of SAP.

## Additional files

Additional file 1: **Methods.** Characterisation of human adipose tissue-derived mesenchymal stem cells; characterisation of pancreatic acinar cells; cell viability assay. (DOCX 21 kb)

Additional file 2: **Figure S1.** Characterisation of human adipose tissue-derived mesenchymal stem cells. (JPG 349 kb)

Additional file 3: **Figure S2.** Characterisation of mouse primary pancreatic acinar cells and caerulein plus LPS stimulation assay. (JPG 242 kb)

Additional file 4: **Table S1.** Histopathological scoring of pancreatic injury; **Table S2.** Primers used for this study. (DOCX 27 kb)

Additional file 5: **Figure S3.** Effects of hAT-MSCs on severe acute pancreatitis (SAP)-associated lung injury in mice. (JPG 454 kb)

## Abbreviations

ATF: Activating transcription factor
CHOP: CCAAT-enhancer-binding protein homologous protein
DMEM: Dulbecco’s modified Eagle’s medium
ELISA: Enzyme-linked immunosorbent assay
ER: Endoplasmic reticulum
FBS: Foetal bovine serum
GAPDH: Glyceraldehyde-3-phosphate dehydrogenase
Grp78: 78-kDa glucose-regulated protein
H&E: Haematoxylin and eosin
hAT-MSCs: Human adipose tissue-derived mesenchymal stem cells
IBD: Inflammatory bowel disease
IL: Interleukin
IRB: Institutional Review Board
IRE: Inositol-requiring enzyme
LPS: Lipopolysaccharide
NF-κB: Nuclear factor kappa B
MSCs: Mesenchymal stem cells
PACs: Pancreatic acinar cells
PBS: Phosphate-buffered saline
PERK: Protein kinase RNA [PKR]-like ER kinase
PGE2: Prostaglandin E2
qRT-PCR: Quantitative reverse transcription polymerase chain reaction
SAP: Severe acute pancreatitis
scr: Scrambled
TNF-α: Tumour necrosis factor alpha
TSG-6: Tumour necrosis factor-α-induced gene/protein 6
