# Supplementary material for: TSG-6 secreted by human adipose tissue-derived mesenchymal stem cells ameliorates severe acute pancreatitis via ER stress downregulation in mice
Source: Stem Cell Res Ther. 2018 Sep 26;9:255. doi: 10.1186/s13287-018-1009-8 (PMC6158864; doi:10.1186/s13287-018-1009-8)
Supplement: Supplementary file 1 — Methods. Characterisation of human adipose tissue-derived mesenchymal stem cells; characterisation of pancreatic acinar cells; cell viability assay. (DOCX 21 kb) [file 13287_2018_1009_MOESM1_ESM.docx]

**Supplementary Methods**

**Characterization of hAT-MSCs**

Stem cells were characterized by several stem cells makers using flow cytometry via fluorescein isothiocyante (FITC)-, or phcoerythrin (PE)-conjugated antibodies against the following proteins: CD31-FITC, CD34-PE, CD45-FITC, CD73-PE, and CD90-PE (all from BD Biosciences, Franklin Lakes, NJ, USA). Stem cells were analyzed using a FACSalibur flow cytometer (BD Biosciences) with the CELL Quest software (BD Biosciences).

**Characterization of PACs**

We separated PACs from C57BL/6 mice using collagenase digestion, and PACs characterized by qRT-PCR analyzed. After the isolation of primary acinar cells, markers of ductal, α-islet, and β-islet cells, namely, CK-19, glucagon, and insulin-1, respectively, were compared between primary acinar cells and pancreatic tissue. Also PACs specific makers including AMY2B, CPA1, and PTF1α were measured by qRT-PCR.

**Cell viability assay**

Pancreatic acinar cells viability on the sample were assessed by D-plus^tm^ Cell Counting Kit (CCK)-8 kit (Dongyinbio, Seoul, Korea). Total 10,000 of the cells were seeded in 96 well plate (SPL Life Science, Pocheon, Korea). After culture for 0, 4, 8, 12, and 24 h, 10 μl of cell counting kit solution was added to each well, and the absorbance was measured at 450 nm.
