# Supplementary figures and images for: TSG-6 secreted by human adipose tissue-derived mesenchymal stem cells ameliorates severe acute pancreatitis via ER stress downregulation in mice
Source: Stem Cell Res Ther. 2018 Sep 26;9:255. doi: 10.1186/s13287-018-1009-8 (PMC6158864; doi:10.1186/s13287-018-1009-8)

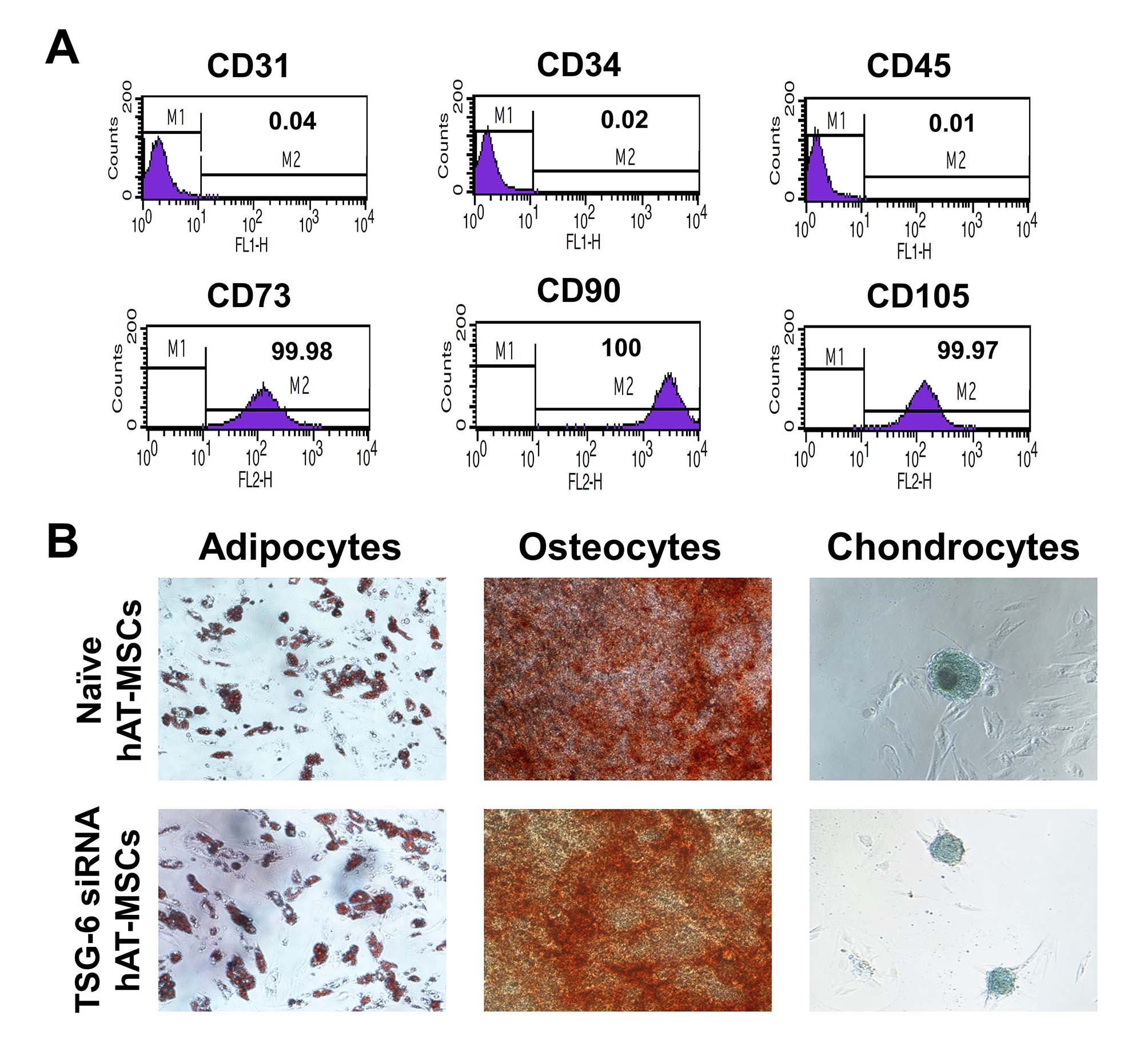

Supplement: Supplementary file 2 — Figure S1. Characterisation of human adipose tissue-derived mesenchymal stem cells. (JPG 349 kb) [file 13287_2018_1009_MOESM2_ESM.jpg]

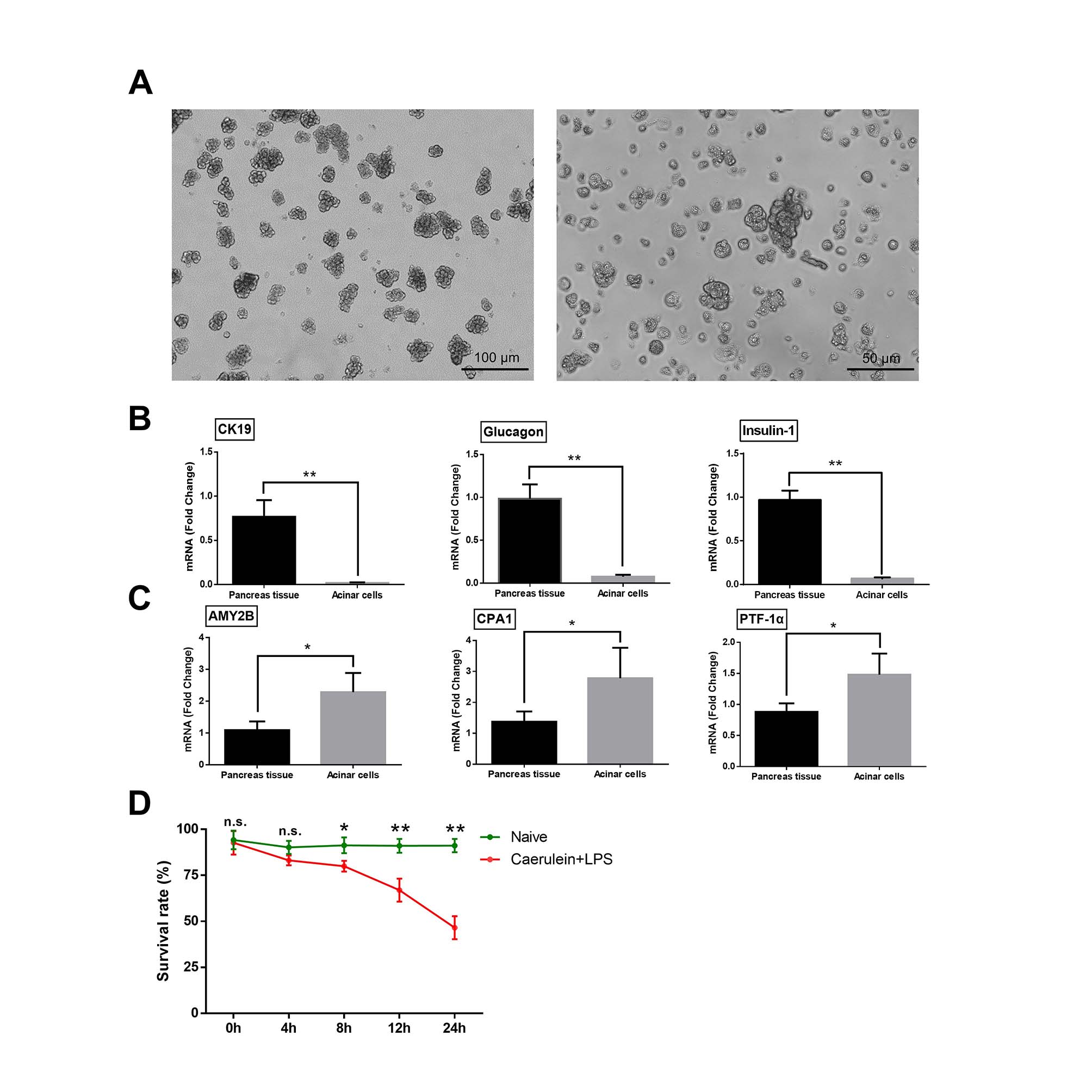

Supplement: Supplementary file 3 — Figure S2. Characterisation of mouse primary pancreatic acinar cells and caerulein plus LPS stimulation assay. (JPG 242 kb) [file 13287_2018_1009_MOESM3_ESM.jpg]

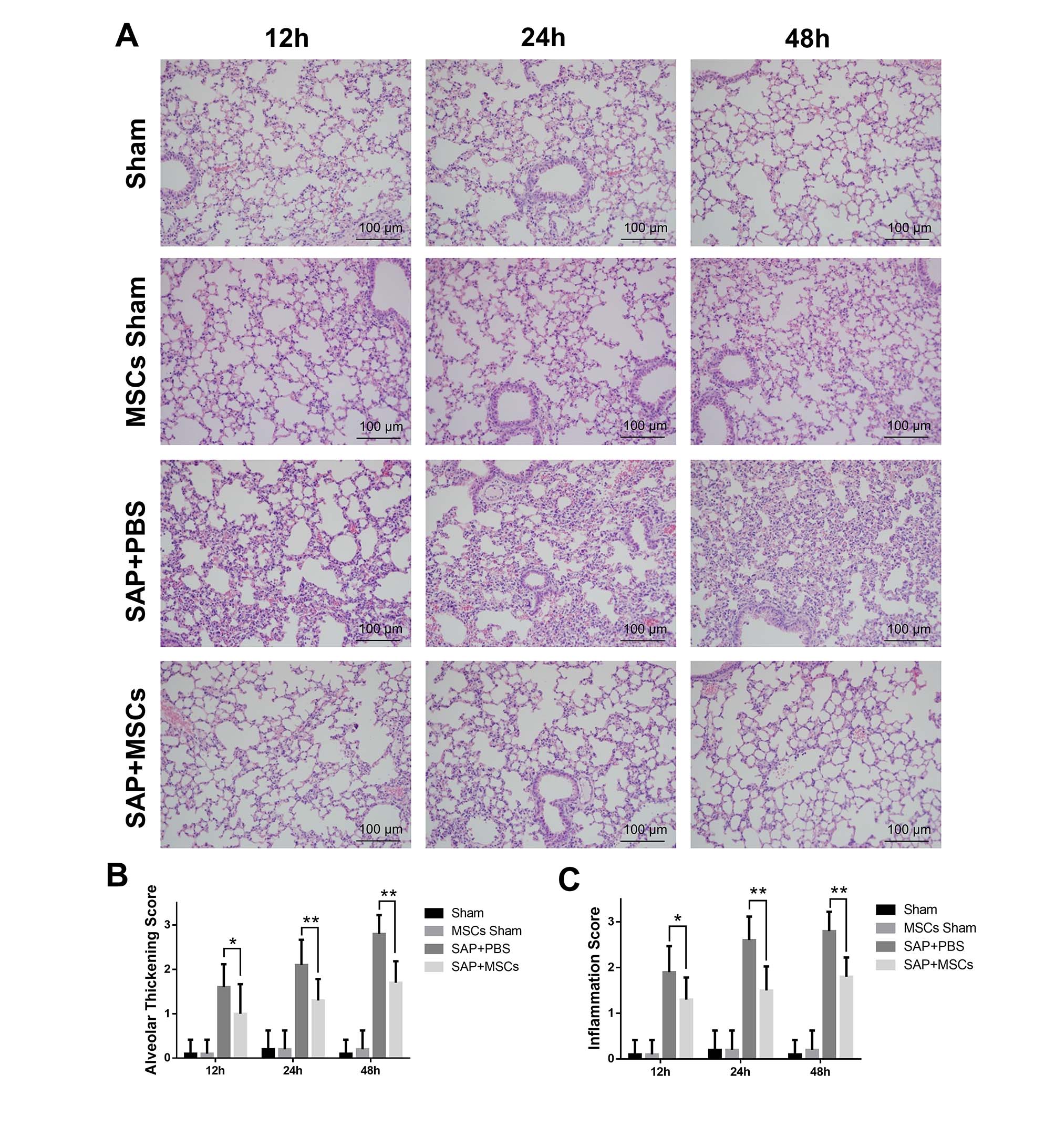

Supplement: Supplementary file 5 — Figure S3. Effects of hAT-MSCs on severe acute pancreatitis (SAP)-associated lung injury in mice. (JPG 454 kb) [file 13287_2018_1009_MOESM5_ESM.jpg]
