# Supplementary material for: TSG-6 secreted by human adipose tissue-derived mesenchymal stem cells ameliorates severe acute pancreatitis via ER stress downregulation in mice
Source: Stem Cell Res Ther. 2018 Sep 26;9:255. doi: 10.1186/s13287-018-1009-8 (PMC6158864; doi:10.1186/s13287-018-1009-8)
Supplement: Supplementary file 4 — Table S1. Histopathological scoring of pancreatic injury; Table S2. Primers used for this study. (DOCX 27 kb) [file 13287_2018_1009_MOESM4_ESM.docx]

**Table S1. Histopathological Scoring of Pancreatic injury.** Inflammatory infiltration and acinar necrosis were counted as the average number per 10 fields at magnification 400X.

| **Score** | **Edema** | **Inflammation** | **Acinar necrosis** |
| --- | --- | --- | --- |
| 0 | Absent | Absent | 0-5 intralobular or perivascular leukocytes/ HPF |
| 1 | Diffuse expansion of interlobar septae | Diffuse occurrence of 1-4 necrotic cells/HPF | 6-15 intralobular or perivascular leukocytes/ HPF |
| 2 | Same as 1 + diffuse expansion of interlobular septae | Diffuse occurrence of 5-10 necrotic cells/HPF | 16-25 intralobular or perivascular leukocytes/ HPF |
| 3 | Same as 2 + diffuse expansion of interacinar septae | Diffuse occurrence of 11-16 necrotic cells/HPF (foci of confluent necrosis) | 26-35 intralobular or perivascular leukocytes/ HPF |
| 4 | Same as 3 + diffuse expansion of intercellular spaces | >16 necrotic cells/HPF (Extensive confluent necrosis) | >35 leukocytes/HPF or confluent microabscesses |

| Gene | Forward (5’-3’) | Reverse (5’-3’) |
| --- | --- | --- |
| **Mouse** |  |  |
| *TNF-α* | CCCTCACACTCAGATCATCTTCT | GCTACGACGTGGGCTACAG |
| *IL-1β* | GTCTTTCCCGTGGACCTTC | TGTTCATCTCGGAGCCTGT |
| *IL-6* | TTATATCCAGTTTGGTAGCATCCAT | AGGCTTAATTACACATGTTCTCTGG |
| *IL-10* | GTGATTTTAATAAGCTCCAAGACCA | GATCATCATGTATGCTTCTATGCAG |
| *Grp78* | TACCAAGTGTAAGGGGACAAAC | ATTCAGCAACTGGTGAAAGAGT |
| *CHOP* | GAAACGAAGAGGAAGAATCAAA | CACTCTGTTTCCGTTTCCTAGT |
| *Caspase12* | GCAATGAAATCTGTCTCCACAT | ACTGTCGGAGTCTGAGAAACAA |
| *GAPDH* | AGTATGTCGTGGAGTCTACTGGTGT | AGTGAGTTGTCATATTTCTCGTGGT |
| *CK19* | GGACCCTCCCGAGATTACAACCA | GCCAGCTCCTCCTTCAGGCTCT |
| *insulin 1* | AGACCTTGGCGTTGGAGGTGGCCCG | GCAGAGGGGTGGGGCGGGTCGAG |
| *glucagon* | ACCTGGACTCCCGCCGTGCCCA | TCGCCTTCCTCGGCCTTTCACCAGCC |
| *PTF1-α* | AGAAGGTTATCATCTGCCATCG | TGGTTCGTTCTCTATGTTGTCG |
| *CPA-1* | TACTTGAACTTGGTCCCGTGTA | GAACATCAAGGCCTTCATCTCC |
| *Amylase 2* | TGGCGTCAAATCAGGAACATGG | GGCTGACAAAGCCCAGTCATCA |
| **Human** |  |  |
| TSG-6 | AAAAACTGGCATTATTGATTATGGA | CAGTAGCAGATTTGGTTATCTTCGT |
| GAPDH | TGCTTTTAACTCTGGTAAAGTGGATA | GTGGAATCATATTGGAACATGTAAAC |

**Table S2. List of primer for qRT-PCR**
